# Individualized Therapy with Ranibizumab in Wet Age-Related Macular Degeneration

**DOI:** 10.1155/2015/412903

**Published:** 2015-09-27

**Authors:** Alfredo García-Layana, Marta S. Figueroa, Luis Arias, Javier Araiz, José María Ruiz-Moreno, José García-Arumí, Francisco Gómez-Ulla, María Isabel López-Gálvez, Francisco Cabrera-López, José Manuel García-Campos, Jordi Monés, Enrique Cervera, Felix Armadá, Roberto Gallego-Pinazo

**Affiliations:** ^1^Clínica Universidad de Navarra, Avenida de Pío XII 36, 31008 Pamplona, Spain; ^2^Sociedad Española de Retina y Vítreo (SERV), C/ Xosé Chao Rego 8, 15705 Santiago de Compostela, Spain; ^3^RETICS OFTARED (RD12/0034) “Prevention Early Detection and Treatment of the Prevalent Degenerative and Chronic Ocular Pathology”, Institute of Health Carlos III, C/ Sinesio Delgado 4, 28029 Madrid, Spain; ^4^Hospital Universitario Ramon y Cajal, Carretera de Colmenar, km 9, 28034 Madrid, Spain; ^5^Vissum Madrid, Santa Hortensia 58, 28002 Madrid, Spain; ^6^Hospital de Bellvitge, C/ Feixa Llarga s/n, L'Hospitalet de Llobregat, 08907 Barcelona, Spain; ^7^Hospital de San Eloy, Avenida Antonio Miranda 5, 48902 Baracaldo, Spain; ^8^Hospital Universitario de Albacete, Avenida de Almansa s/n, 02006 Albacete, Spain; ^9^Hospital Vall D'Hebron, Passeig de la Vall d'Hebron 119–129, 08035 Barcelona, Spain; ^10^Instituto Oftalmológico Gómez-Ulla, Calle Maruja Mallo 3, 15706 Santiago de Compostela, Spain; ^11^IOBA, Hospital Clínico Universitario de Valladolid, Paseo de Belén 17, 47011 Valladolid, Spain; ^12^Complejo Hospitalario Universitario Insular Materno Infantil de Gran Canaria, Avenida Marítima del Sur s/n, 35016 Las Palmas de Gran Canaria, Spain; ^13^Hospital Universitario Virgen de la Victoria, Campus de Teatinos s/n, 29010 Málaga, Spain; ^14^Institut de la Macula i de la Retina, Carrer de Vilana 12, 08022 Barcelona, Spain; ^15^Hospital General de Valencia, Avenida Tres Cruces 2, 46014 Valencia, Spain; ^16^Hospital la Paz, Paseo de la Castellana 261, 28046 Madrid, Spain; ^17^Hospital la Fe, Avenida de Fernando Abril Martorell 106, 46026 Valencia, Spain

## Abstract

Individualized treatment regimens may reduce patient burden with satisfactory patient outcomes in neovascular age-related macular degeneration. Intravitreal anti-VEGF drugs are the current gold standard. Fixed monthly injections offer the best visual outcome but this regimen is not commonly followed outside clinical trials. A PRN regimen requires monthly visits where the patient is treated in the presence of signs of lesion activity. Therefore, an early detection of reactivation of the disease with immediate retreatment is crucial to prevent visual acuity loss. Several trials suggest that “treat and extend” and other proactive regimens provide a reasonable approach. The rationale of the proactive regimens is to perform treatment anticipating relapses or recurrences and therefore avoid drops in vision while individualizing patient followup. Treat and extend study results in significant direct medical cost savings from fewer treatments and office visits compared to monthly treatment. Current data suggest that, for one year, PRN is less expensive, but treat and extend regimen would likely be less expensive for subsequent years. Once a patient is not a candidate to continue with treatment, he/she should be sent to an outpatient unit with adequate resources to follow nAMD patients in order to reduce the burden of specialized ophthalmologist services.

## 1. Early Diagnosis and Treatment Initiation

Age-related macular degeneration (AMD) is the leading cause of blindness among the elderly in the Western world [1–3]. Currently, there is no cure for the disease; however, intravitreal antivascular endothelial growth factor (anti-VEGF) agents have significantly improved visual outcomes in patients with neovascular age-related macular degeneration (nAMD) [4–7]. These new therapeutic approaches have been shown to prevent and in some cases reverse visual damage caused by nAMD in clinical trials. Early diagnosis is obviously essential in order to take action as promptly as possible to obtain the best result from therapy [8]. Therefore, primary care physicians who suspect nAMD should directly refer their patients to an ophthalmologist [9]. It is advisable to establish a referral protocol based on signs and symptoms in order to maximize efficiency and utilization of health resources.

Based on clinical evidence, most protocols recommend administering three consecutive monthly intravitreal injections of ranibizumab [8, 10, 11]. The pivotal studies MARINA [4] and ANCHOR [5] and later Pronto [12], SUSTAIN [13], and IVAN [14] scheduled three loading doses of ranibizumab as initial treatment. Their results have shown that visual acuity (VA) improves plateaus after the first three injections. The current summary of product characteristics of ranibizumab recommends initiating the treatment with a loading phase consisting of a monthly injection of 0.5 mg ranibizumab during three consecutive months. However, more recently CATT [6] protocol found that after the first year ranibizumab given as needed without the use of three mandatory loading doses was equivalent to ranibizumab given monthly. Therefore, although most clinical protocols recommend a loading phase, we still do not have conclusive data to support the superiority of three mandatory monthly initial doses over one dose.

These results have also been endorsed recently with the use of aflibercept in the VIEW-1 and VIEW-2 studies which have established its indication for the treatment of nAMD with a recommended regimen of 2 mg injection every 8 weeks during the first year following a loading phase of three injections [7].

Furthermore, the response to the initial loading dose constitutes a very important parameter to assess the possible progression of the patient, to establish a profile for future response to treatment, and to individualize the therapy [15, 16]. Thus, following the loading dose in the SUSTAIN study, 53% maintain what was gained in the first three months, 21% do not maintain it, and 26% did not gain vision [13].

As far as bevacizumab is concerned, some studies also recommend a loading dose [17, 18], but in CATT study, the comparison between bevacizumab given as needed without loading phase and bevacizumab given monthly was inconclusive, so neither no inferiority nor inferiority was established between the two study groups [6].

## 2. Individualized Treatment Protocols

Intravitreal ranibizumab based on a PRN (*Pro Re Nata* or “as needed”) regimen where retreatment is given in the presence of signs of activity is frequently used for the management of neovascular age-related macular degeneration. However, strict monthly monitoring is required to obtain the best results. This represents a huge burden for both the ophthalmologist and the patient, though some patients do not need to be monitored monthly. Individualization of the treatment and followup is a key to ensure optimal clinical outcomes for those patients.

In the pivotal MARINA and ANCHOR trials, ranibizumab was administered monthly for two years in patients with nAMD. With this treatment protocol, mean VA improved 6.6 letters and 10.7 letters, respectively [4, 5]. In the VIEW trials, bimonthly aflibercept 2.0 mg showed very similar visual results compared to monthly ranibizumab 0.5 mg [7].

The Pronto study pioneered retreatment based on optical coherence tomography (OCT) findings. In this study, 40 patients were monitored monthly after receiving a loading dose of three consecutive ranibizumab injections. Retreatment was mainly administered in the presence of VA loss and persistent or recurrent intraretinal or subretinal fluid on OCT scans. At 24 months, mean VA improved 11 letters and 43% of patients gained 15 or more letters, with an average of 9.9 injections [12]. However, the promising results of the Pronto study could not be reproduced in the larger SUSTAIN trial with a similar treatment regimen [13]. These studies laid the foundations of the so-called PRN regimen. Notwithstanding, strict monthly monitoring is required to achieve a good clinical outcome with no tolerance to the presence of macular fluid detected on OCT, which has been demonstrated in the CATT trial [6]. The CATT study concluded that ranibizumab and bevacizumab were equivalent in terms of VA results when given under the same treatment protocol; however, ranibizumab better corrected anatomic pathologies as demonstrated by OCT. At two years, monthly injections of each treatment were found to be superior to their respective PRN regimens. Importantly, this study has also shown that patients receiving monthly injections with either ranibizumab or bevacizumab during the first year who are then rerandomized to PRN treatment during the second year had similar VA results as patients treated with a PRN regimen from the start. Therefore, an aggressive approach at the beginning of the disease does not seem to be a key factor for a better long-term outcome.

It seems that the key to the success of monthly dosing may possibly lie in treating before further disease progression, that is, treatment being administered even when the lesion is inactive. In this approach, the treatment has the advantage—not the disease. If this theory was correct, the ideal treatment would be one that anticipates and prevents disease but at the same time avoids the burden and risks of the monthly injections. The proactive regimens such as treat and extend or the Fusion regimens seek this goal.

In the “inject and extend” or “treat and extend” regimens, the intervals between reinjections are progressively extended if there is no fluid, thereby “titrating” a patient's individual maximal treatment-free interval [19, 20]. The visual results of these regimens gave satisfactory results. The 1-year results of the LUCAS trial, the largest and best performed treat and extend study, showed equivalent effectiveness in the VA scores with ranibizumab (8.2 letters) and bevacizumab (8 letters). These results were achieved with a mean of eight injections in the ranibizumab group and a mean of 8.8 injections in the bevacizumab group. The difference in the number of injections reached significance (*P* = 0.002) [19]. In addition, a small retrospective nonrandomized case series showed more favourable results with the treat and extend regimen than with PRN, probably because patients in the PRN group were only examined 8.8 times a year instead of the 12 times initially expected [20]. According to the ASRS PAT survey, treat and extend regimen is preferred by most physicians (communication at the American Academy Meeting in New Orleans, November 2013). In the treat and extend regimen, the patients are treated regardless of the presence of fluid or neovascular activity. In the absence of neovascular activity, the patient receives the treatment at each visit; however, the interval between subsequent visits will continually be extended several weeks unless disease activity returns. In this manner, the treatment anticipates and avoids the relapses. In the case of the PRN regimen, the treatment tries to catch up to the relapses but has no chance by definition to prevent them. Randomized prospective studies comparing the two methods are now needed to establish the best reinjection strategy for patients treated with anti-VEGF drugs, as the one currently in progress sponsored by the Spanish Vitreoretinal Society (*In Eye study*, EudraCT number 2012-003431-37).

The Fusion regimen combines the PRN approach with fixed injections after certain periods of (apparent) inactivity [21, 22]. In the second year of the VIEW study, a similar approach was followed and was termed capped-PRN [7]. The Fusion regimen consists of three steps. First, a loading phase of three consecutive monthly injections is given. If CNV activity is resolved at the first follow-up visit, the loading phase can be reduced to two monthly injections. Second, a PRN regimen is established on demand, and intravitreal injections are given if CNV activity is present. After cessation of CNV activity, patients still receive one injection. Third, after cessation of CNV activity, patients receive fixed injections every 2 months for two courses and every 3 months for two courses. At the intermediate visits, between the preplanned fixed injections, patients are treated according to PRN criteria [22].

Though conceptually different, the Fusion regimen has some similarities with the proposed “treat and extend” regimen [23, 24] because both regimens aim at the prevention of disease recurrence. The main differences between the Fusion and “treat and extend” regimens are that in the latter the periods between treatments are extended in a continuous linear form and that there are no visits in between these periods.

In summary, the rationale of the proactive regimens is to perform treatment anticipating the relapses or recurrences and therefore avoid drops in vision while individualizing patient followup. The objective is to avoid irreversible loss of vision at recurrences despite reactive treatment. Proactive regimens not only reduce the number of injections and their associated risks as do PRN regimens but also minimize the number of visits and their associated costs and inconvenience. Treat and extend has the potential to be most cost-effective regimen, by reducing the frequency of injections compared to monthly regimen, as well as the number of visits compared to PRN. Due to the proactivity of the treatment, this reduced cost does not compromise clinical benefit or efficacy.

The main weakness so far is that these regimens have not yet been validated in larger, controlled, randomized trials.

## 3. Socioeconomical Burden

Different challenges must be faced in the long-term treatment of nAMD with intravitreal injections of ranibizumab. One of the major problems is how to manage a high number of patients who require frequent monitoring visits and retreatments. It is known that monthly visits are mandatory during the initial period after the onset of the disease to optimize the results that can be obtained with the treatment. However, these follow-up visits can be progressively extended if the lesion remains stable. Currently, there is lack of evidence to recommend a “treat and extend” regimen instead of a PRN regimen. The percentage of patients gaining 15 or more letters and the mean VA change are not as good in studies performed in a “real-world” context as in clinical trials. This could be due to the wide range of lesions treated in clinical practice compared to the strictly selected ones in randomized trials. Furthermore, although flexible regimens may reduce the burden of visits and injections, they might offer a less favourable long-term outcome.

The goal of OCT-driven treatment protocols is to achieve a “dry macula” with repeated injections. Nevertheless, some residual fluid may be left untreated if VA is stable during several follow-up visits. This might be relevant to save some extra injections making the treatment more cost-effective.

## 4. Signs of Activity and Retreatment Criteria in Individualized Therapies

Initially, it should be established that there is no structural damage defined as longstanding fibrosis or atrophy in the foveal area, significant chronic disciform scar, or other severe ocular diseases like vitreous hemorrhage or rhegmatogenous retinal detachment. It is also recommended that treatment with ranibizumab should not be commenced if there is evidence or suspicion of hypersensitivity to this drug [11].

The choroidal neovascular membrane should be considered active if it presents any of the following features: abnormal retinal thickness, particularly if there is evidence of accumulated intraretinal or subretinal fluid, or fluid under the RPE, confirmed by OCT; presence or recurrence of intraretinal fluid and/or subretinal fluid or subretinal hemorrhage; new or persistent leakage detected with angiography fluorescein; choroidal neovascular membrane growth detected with angiography fluorescein; and VA deterioration, considered to represent deterioration of the choroidal neovascular membrane [25].

Choroidal neovascular membrane disease progression is defined by the Institute for Health and Clinical Excellence [26] as the appearance of sight-threatening choroidal neovascular membrane which was not previously suspected or thought to be present or evidence of new hemorrhage and/or subretinal fluid or a documented recent visual decline in the choroidal neovascular membrane or an increase in the lesion size between visits.

The disease should be considered to have become inactive when there is [11] persistence or absence of fluid with absence of fluorescein leakage or other signs of disease activity like increasing lesion size, new haemorrhages, or exudates; no deterioration in vision that can be attributed to choroidal neovascular membrane activity; no lesion growth or new signs of disease activity on subsequent followup following recent discontinuation of treatment; and no worsening of OCT indicators of choroidal neovascular membrane disease.

Retreatment criteria from Pronto study [12] are defined if any of the following changes were observed: VA loss of at least 5 letters or 1 EDTRS line; an increase in OCT of at least 100 m; new macular hemorrhage; new area of classic neovascular choroidal membrane; or evidence of persistent fluid on OCT one month after injection. Detachment of pigment epithelium was recorded as an OCT finding but was not included as retreatment criteria. This decision was based on the observations from phase I/II extension Pronto study with ranibizumab. In this study, little correlation between the presence of pigment epithelium detachment and VA was observed. During the second year of the study, retreatment criteria were changed to include any qualitative modifications in the OCT that suggested fluid in the macular area. These changes included the appearance of retinal cysts or subretinal fluid or an enlargement of the pigment epithelium detachment. Any of these qualitative changes alone was sufficient to permit retreatment [12].

In the SUSTAIN [13] study, retreatment was based on VA and OCT findings indicating loss of more than five letters of VA from the previous highest VA score and OCT criteria ≥100 m increase from the previous lowest measurement.

It is important to remember that some degenerative retinal changes observed with OCT can be presented as hyporeflective images and may not correspond to fluid but structural changes like the residual internal layers pseudocysts or the outer retinal tabulations [27]. In these cases we must rule out the possibility that the fluid is not representing disease activity.

## 5. Nonresponder and Therapeutical Approach

There is not an accepted definition of nonresponders to nAMD treatment. However, in clinical practice, some patients do not respond adequately to the treatment. Even with monthly injections of ranibizumab at the 2-year followup, 9% of the patients in the MARINA study and 10% of patients in the ANCHOR study lost more than 15 letters of VA [28]. Patients with good VA at baseline might have difficulty improving 3 lines of VA compared with the greater likelihood of losing 3 lines of VA. The opposite is likely true for patients with poor VA at baseline. It may be impossible for a patient with poor VA at baseline to lose an additional 3 lines of VA, whereas there is a greater likelihood of improving 3 lines once treatment is initiated [28]. If vision loss is associated with a suppressed CNV without leakage but is associated with pigmentary abnormalities and geography atrophic scar, it does not seem that a shift to another treatment would allow any improvement.

In addition, the failure of anti-VEGF monotherapy to provide a long-lasting resolution of intraretinal or subretinal fluid is frequently observed even in clinical trials. In the CATT study, the proportion of patients without fluid on OCT in the ranibizumab monthly group, where the best anatomical results were obtained, was 45.5% after two years of followup [6].

Therefore, both functional and anatomical aspects must be considered when we define a nonresponder, and we must consider both the short time and the long time after beginning the treatment, in order to decide the management of these patients.

In the short term, the SUSTAIN study identified three groups of responders to ranibizumab therapy after the loading doses [13]: those who gained vision (one or more letters) in the initial 3 months and maintained that vision in the maintenance phase (53%); those who gained vision in the initial 3 months but did not maintain it through the maintenance phase and stabilized at the baseline VA level (21%); and those who had no initial vision gain and no gain during the PRN phase. In that group, rather a decrease in BCVA was observed during the follow-up period (26%). In this third group, if VA gets worse and fluid is still present in the OCT, we can consider the patient a “real nonresponder.” If the patient has a bad VA and a disciform scar, probably no additional treatment should be provided, although this can be reconsidered if it is the eye with the best VA of the patient.

If the patient has still a useful vision, additional tests should be used to determine different macular diseases that should be treated in a different way. Diagnosis of polypoidal choroidal vasculopathy (PCV) should be based on early phase hyperfluorescent hot spots visualized using indocyanine green angiography (ICG). Treatment of PCV is usually performed with combined therapy using ICG-guided verteporfin photodynamic therapy plus three doses of ranibizumab intravitreal injections 1 month apart [29].

Differentiating long-standing central serous chorioretinopathy (CSC) from nAMD can sometimes be difficult. Chronic CSC patients often develop in old patients, with RPE hyperplasia or atrophy, subretinal fluid and cystoid edema in the OCT, and hyperfluorescence on fluorescein and ICG. CSC can also be diagnosed with the help of enhanced depth imaging on OCT. In addition, some patients presenting with choroidal neovascularization have a missed background of CSC and the etiology is erroneously ascribed to nAMD. Atrophic changes in RPE are better seen with fundus autofluorescence imaging and midphase. ICG showing bilateral choroidal hyperpermeability can also help with the diagnosis. In the OCT, subretinal fluid and hypertrophic outer retinal changes are more common but intraretinal fluid is less frequent. Choroidal thickness is also increased in CSC. Chronic CSC is better treated with ICG-guided verteporfin photodynamic therapy and ranibizumab may be also added if CNV has also complicated the process [30].

Those who gained vision in the initial 3 months but did not maintain it must be considered “responders” although the type of response is not so good. Some studies suggest that patients treated with repeat intravitreal bevacizumab and ranibizumab injections may demonstrate tachyphylaxis over time [31]. When a patient does not respond adequately to the drug of choice, the question arises whether a shift in therapy to another VEGF inhibitor will be useful. Shifting patients to ranibizumab after insufficient response to bevacizumab has shown that most patients improved the anatomic status of the macula on OCT (32% complete resolution of fluid and 39% with partial response). However, the mean VA did not change after shifting treatment, with individual changes ranging from loss of 4 lines to gain of 2 lines [32]. When patients are transitioned from bevacizumab, ranibizumab, or both to aflibercept, 7% gained 2 lines or more and 85% showed stable VA. Again, an improvement in the anatomic outcomes was observed in the OCT after the change to aflibercept with 5% showing complete resolution of baseline exudative fluid and 49% showing partial resolution [33].

Finally, another group of responders are those who gain and maintain visual improvement but need almost continuous monthly injections, because there is always fluid in the OCT during the follow-up visits. As previously mentioned, in these cases we must rule out the possibility that the fluid is not representing disease activity, like the residual internal layers pseudocysts or the outer retinal tabulations [27]. In addition, a follow-up visit may be scheduled two weeks after the intravitreal injection in order to know if a patient has an initial improvement of the fluid after treatment, which then rapidly recurs with one month's time [34]. In these cases, treatment every two weeks, change of anti-VEGF drug, or combined therapy with verteporfin and anti-VEGF may be postulated [23, 33, 34]. Recently, some steroids and slow-delivered steroid implants combined with anti-VEGF have been postulated as an alternative option in eyes with refractory nAMD [24, 35].

## 6. Criteria for Patient Follow-Up Externalization

Interruption of the treatment can be considered in two ways: temporary suspension of anti-VEGF injections whilst still maintaining periodic controls or definite interruption of both the injections and patient followup [36].

Just as in one way or another, the Pronto study has shown that in certain cases the treatment with ranibizumab can be suspended, at least temporarily without showing a loss of the benefits obtained [12]. At the same time, it is observed that up to 30% of the cases did not require additional treatment [36].

Meanwhile, the decision of suspending anti-VEGF treatment in nAMD may result in very different situations. In favourable situations, the treatment is interrupted due to the long-term resolution of exudation and stability of vision; in unfavourable situations, the interruption is fundamentally due to the development of a fibrotic macular disciform scar, chronic macular edema, or macular atrophy. The SEVEN-UP study has shown that, approximately 7 years after ranibizumab therapy, one-third of patients demonstrated good visual outcomes, whereas another third had poor outcomes. Active exudative disease was detected by spectral-domain OCT in 68% of study eyes, and 46% were receiving ongoing ocular anti-VEGF treatments. Macular atrophy was detected by fundus autofluorescence in 98% of eyes and the area of atrophy correlated significantly with poor visual outcome [37].

For these reasons, we can conclude by affirming that once the patient with nAMD is not a candidate to continue with treatment, he/she should be sent to AMD outpatients units, where a periodic followup can be carried out by a specialist in macular disease who can perform the following tests: measurement of VA, fundus examination, and macular OCT [8]. At the same time, the aforementioned could be complimented with the referral of certain patients to low vision units (teams formed by ophthalmologists, opticians, rehabilitation technicians, social workers, and psychologists), thus increasing the use of units that are underused at present [38]. All of this would contribute to reducing the healthcare burden in hospitals and assuring a satisfactory control of nAMD patients.

The creation of an AMD outpatient unit would therefore signify a clear advance in assuring a better quality of life for a chronic patient and reducing the healthcare burden of the ophthalmologist services in an illness that has a strong tendency for periodic visits (closer periods versus spaced out checkups) and a growing demand due to demographic factors [39].

## 7. Real-Life Practice Results of Anti-VEGF in nAMD

Apart from clinical trials, several studies have explored different regimens for the treatment of nAMD with ranibizumab in the “real world.” Most of them are based on a PRN scheme but with more flexible criteria than in clinical trials to reduce the burden of injections and follow-up visits [40]. However, overall results of these studies are not as good as those obtained following the more rigid protocols of clinical trials. The PRN regimens performed outside clinical trials have many potential sources of noncompliance. Therefore, the risk of an even worse outcome is higher and it remains controversial whether initial good results can be maintained over time [41]. Therefore, despite the ophthalmology community's joy at new developments in the treatment of nAMD in 2005 [4–7], the inconvenience of monthly injections or at least monthly followup remained. In addition, when analysing different patient responses across clinical trials, individualization of the treatment and the followup is needed. In the Lumiere study performed in France, the mean VA gain at 12 months was 3.2 ± 14.8 ETDRS letters [42]. Fewer than 40% of patients received the recommended treatment of initial 3 monthly injections, 50% patients had to wait more than 8 days for the initial anti-VEGF treatment, and the average number of injections was 5.1 during the 12-month period.

The retrospective pooled analysis of four European registries (Wave/Germany; Helios/Netherlands; Helios/Belgium; Sweden) within the LUMINOUS program has shown that the mean improvement in ETDRS letters at 12 months was 0, 5.6, 2.5, and 1 with a mean number of 4.3, 5.5, 5.0, and 4,7 injections, respectively [43].

In the AURA multinational study, the mean improvement from baseline in France was 0.8 letters and 0.1 at 1 year and 2 years, respectively [44]. In Germany, these figures were −0.4 and −2.4, respectively. The mean number of injections in years 1 and 2 was 4.4 and 1.9 in France and 4.2 and 1.1 in Germany [44].

In Spain, a study has evaluated the degree of compliance by Spanish retinal specialists with the Spanish Vitreoretinal Society Guidelines for Management of AMD [45]. This multicenter retrospective observational study included 346 patients. Adherence to SERV guidelines was high for the diagnosis (96,8%), medium for initial treatment (84,4%), and low for followup and retreatment (46,9%). In the first year, follow-up visits were made every 2 months or more frequently in 66.2% of patients. In the second year, followup was even less frequent: every 3 months or more in 70.2% of patients.

Another study in 12 sites across Spain, which included 208 patients followed for 24 months, has shown that the average number of follow-up visits was 9 (5.4 and 3.6 during the first and second year), the mean number of injections was 6.1 (4.5 and 1.6 during the first and second year), and the mean VA gain was 2.4 ± 16.6 letters at 12 months and 3.1 ± 19.6 at 24 months [46].

Treatment with intravitreal anti-VEGF injections in real-life practice produces, on average, poorer-than-expected visual outcomes, probably due to fewer injections per year and less than monthly monitoring. Recently, a Delphi study to detect deficiencies in real-life treatment of nAMD was performed in one hundred members of the Spanish Vitreoretinal Society [47]. Recommendations were developed after analyzing the differences between the results and the SERV guidelines recommendations. Consensus statements to reduce the burden of the disease included the use of treat and extend regimen and reducing the amount of diagnostic tests during the loading phase and training technical staff to perform these tests and reducing the time between relapse detection and reinjection, as well as establishing patient referral protocols to outside general ophthalmology clinics.

In conclusion, ranibizumab demonstrates a favourable clinical effectiveness and dramatically improves outcomes in nAMD, although some actions must be implemented in order to enhance results in real-world practice. The positioning of ranibizumab in nAMD will be defined more accurately in the future, when data for existing and new therapies become available.

## References

[1] Klein R., Klein B. E. K., Tomany S. C., Moss S. E. (2002). Ten-year incidence of age-related maculopathy and smoking and drinking: the Beaver Dam Eye study. *The American Journal of Epidemiology*.

[2] Sainz-Gómez C., Fernández-Robredo P., Salinas-Alamán Á. (2010). Prevalence and causes of bilateral blindness and visual impairment among institutionalized elderly people in Pamplona, Spain. *European Journal of Ophthalmology*.

[3] Ambati J., Ambati B. K., Yoo S. H., Ianchulev S., Adamis A. P. (2003). Age-related macular degeneration: etiology, pathogenesis, and therapeutic strategies. *Survey of Ophthalmology*.

[4] Rosenfeld P. J., Brown D. M., Heier J. S. (2006). Ranibizumab for neovascular age-related macular degeneration. *The New England Journal of Medicine*.

[5] Brown D. M., Kaiser P. K., Michels M. (2006). Ranibizumab versus verteporfin for neovascular age-related macular degeneration. *The New England Journal of Medicine*.

[6] Martin D. F., Maguire M. G., Ying G.-S., Grunwald J. E., Fine S. L., Jaffe G. J. (2011). Ranibizumab and bevacizumab for neovascular age-related macular degeneration. *The New England Journal of Medicine*.

[7] Trichonas G., Kaiser P. K. (2013). Aflibercept for the treatment of age-related macular degeneration. *Ophthalmology and Therapy*.

[8] Ruiz-Moreno J. M., Arias-Barquet L., Armadá-Maresca F. (2009). Guidelines of clinical practice of the SERV: treatment of exudative age-related macular degeneration (AMD). *Archivos de la Sociedad Espanola de Oftalmologia*.

[9] Bressler N. M. (2002). Early detection and treatment of neovascular age-related macular degeneration. *The Journal of the American Board of Family Practice*.

[10] Cruess A. F., Berger A., Colleaux K. (2012). Canadian expert consensus: optimal treatment of neovascular age-related macular degeneration. *Canadian Journal of Ophthalmology*.

[11] Amoaku W. (2009). Ranibizumab: the clinician's guide to commencing, continuing, and discontinuing treatment. *Eye*.

[12] Lalwani G. A., Rosenfeld P. J., Fung A. E. (2009). A variable-dosing regimen with intravitreal ranibizumab for neovascular age-related macular degeneration: year 2 of the PrONTO study. *The American Journal of Ophthalmology*.

[13] Holz F. G., Amoaku W., Donate J. (2011). Safety and efficacy of a flexible dosing regimen of ranibizumab in neovascular age-related macular degeneration: the SUSTAIN study. *Ophthalmology*.

[14] Chakravarthy U., Harding S. P., Rogers C. A. (2013). Alternative treatments to inhibit VEGF in age-related choroidal neovascularisation: 2-year findings of the IVAN randomised controlled trial. *The Lancet*.

[15] Menghini M., Kurz-Levin M. M., Amstutz C. (2010). Response to ranibizumab therapy in neovascular AMD. An evaluation of good and bad responders. *Klinische Monatsblätter für Augenheilkunde*.

[16] Shin H. J., Chung H., Kim H. C. (2013). Correlation of foveal microstructural changes with vision after anti-vascular endothelial growth factor therapy in age-related macular degeneration. *Retina*.

[17] Bidot M. L., Malvitte L., Bidot S., Bron A., Creuzot-Garcher C. (2011). Etude preliminaire sur l'efficacité de trois injec-tions intravitreennes de bevacizumab dans le traitement de la degenrescence maculaire lieé à l'agê exsudative. *Journal Français d'Ophtalmologie*.

[18] Mekjavic P. J., Kraut A., Urbancic M., Lenassi E., Hawlina M. (2011). Efficacy of 12-month treatment of neovascular age-related macular degeneration with intravitreal bevacizumab based on individually determined injection strategies after three consecutive monthly injections. *Acta Ophthalmologica*.

[19] Berg K., Pedersen T. R., Sandvik L., Bragadóttir R. (2015). Comparison of ranibizumab and bevacizumab for neovascular age-related macular degeneration according to LUCAS treat-and-extend protocol. *Ophthalmology*.

[20] Oubraham H., Cohen S. Y., Samimi S. (2011). Inject and extend dosing versus dosing as needed: a comparative retrospective study of ranibizumab in exudative age-related macular degeneration. *Retina*.

[21] Monés J. (2011). A review of ranibizumab clinical trial data in exudative age-related macular degeneration and how to translate it into daily practice. *Ophthalmologica*.

[22] Monés J., Biarnés M., Trindade F., Casaroli-Marano R. (2012). FUSION regimen: ranibizumab in treatment-naïve patients with exudative age-related macular degeneration and relatively good baseline visual acuity. *Graefe's Archive for Clinical and Experimental Ophthalmology*.

[23] Seidel G., Werner C., Weger M., Steinbrugger I., Haas A. (2013). Combination treatment of photodynamic therapy with verteporfin and intravitreal ranibizumab in patients with retinal angiomatous proliferation. *Acta Ophthalmologica*.

[24] Calvo P., Ferreras A., Al Adel F., Wang Y., Brent M. H. (2014). Dexamethasone intravitreal implant as adjunct therapy for patients with wet age-related macular degeneration with incomplete response to ranibizumab. *British Journal of Ophthalmology*.

[25] Mitchell P., Korobelnik J.-F., Lanzetta P. (2010). Ranibizumab (Lucentis) in neovascular age-related macular degeneration: evidence from clinical trials. *British Journal of Ophthalmology*.

[26] Amoaku W. M. K. (2008). The Royal College of Ophthalmologists interim recommendations for the management of patients with age-related macular degeneration. *Eye*.

[27] Zweifel S. A., Engelbert M., Laud K., Margolis R., Spaide R. F., Freund K. B. (2009). Outer retinal tubulation a novel optical coherence tomography finding. *Archives of Ophthalmology*.

[28] Rosenfeld P. J., Shapiro H., Tuomi L., Webster M., Elledge J., Blodi B. (2011). Characteristics of patients losing vision after 2 years of monthly dosing in the phase III ranibizumab clinical trials. *Ophthalmology*.

[29] Koh A. H. C., Chen L.-J., Chen S.-J. (2013). Polypoidal choroidal vasculopathy: evidence-based guidelines for clinical diagnosis and treatment. *Retina*.

[30] Stangos A. N., Gandhi J. S., Nair-Sahni J., Heimann H., Pournaras C. J., Harding S. P. (2010). Polypoidal choroidal vasculopathy masquerading as neovascular age-related macular degeneration refractory to ranibizumab. *The American Journal of Ophthalmology*.

[31] Schaal S., Kaplan H. J., Tezel T. H. (2008). Is there tachyphylaxis to intravitreal anti-vascular endothelial growth factor pharmacotherapy in age-related macular degeneration?. *Ophthalmology*.

[32] De Geus S. J. R., Jager M. J., Luyten G. P. M., Dijkman G. (2013). Shifting exudative age-related macular degeneration patients to ranibizumab after insufficient response to bevacizumab. *Acta Ophthalmologica*.

[33] Ho S. Y., Yeh S., Olsen T. W. (2013). Short-term outcomes of aflibercept for neovascular age-related macular degeneration in eyes previously treated with other vascular endothelial growth factor inhibitors. *American Journal of Ophthalmology*.

[34] Stewart M. W., Rosenfeld P. J., Penha F. M. (2012). Pharmacokinetic rationale for dosing every 2 weeks versus 4 weeks with intravitreal ranibizumab, bevacizumab, and aflibercept (vascular endothelial growth factor Trap-eye). *Retina*.

[35] Ranchod T. M., Ray S. K., Daniels S. A., Leong C. J., Ting T. D., Verne A. Z. (2013). LUCEDEX: a prospective study comparing ranibizumab plus dexamethasone combination therapy versus ranibizumab monotherapy for neovascular age-related macular degeneration. *Retina*.

[36] Gaudric A., Cohen S. Y. (2010). When should anti-vascular endothelial growth factor treatment be stopped in age-related macular degeneration?. *The American Journal of Ophthalmology*.

[37] Rofagha S., Bhisitkul R. B., Boyer D. S., Sadda S. R., Zhang K. (2013). Seven-year outcomes in ranibizumab-treated patients in ANCHOR, MARINA, and HORIZON: a multicenter cohort study (SEVEN-UP). *Ophthalmology*.

[38] Monés J., Gómez-Ulla F. (2005). *Degeneración Macular Asociada a la Edad*.

[39] Arias L. (2010). *Actualización de Terapia Anti-VEGF en las Enfermedades de la Retina y Coroides*.

[40] Arias L., Roman I., Masuet-Aumatell C. (2011). One-year results of a flexible regimen with ranibizumab therapy in macular degeneration: relationship with the number of injections. *Retina*.

[41] Singer M. A., Awh C. C., Sadda S. (2012). HORIZON: an open-label extension trial of ranibizumab for choroidal neovascularization secondary to age-related macular degeneration. *Ophthalmology*.

[42] Cohen S. Y., Mimoun G., Oubraham H. (2013). Changes in visual acuity in patients with wet age-related macular degeneration treated with intravitreal ranibizumab in daily clinical practice: the lumiere study. *Retina*.

[43] Mitchel P. Ranibizumab in age-related macular degeneration: a pooled analysis of European registries included in the LUMINOUS program.

[44] Hoyng C. B., AURA Steering Committee Retrospective analysis of the real world utilization of ranibizumab in AMD.

[45] López J. D. Adherencia a las recomendaciones de la SERV para el manejo de pacientes con DMAE exudativa por parte de los especialistas retinologos.

[46] Casaroli-Marano R., Gallego-Pinazo R., Fernández-Blanco C. T. (2014). Age-related macular degeneration: clinical findings following treatment with antiangiogenic drugs. *Journal of Ophthalmology*.

[47] García-Layana A., Arias L., Figueroa M. S. (2014). A Delphi study to detect deficiencies and propose actions in real life treatment of neovascular age-related macular degeneration. *Journal of Ophthalmology*.

